# Infective endocarditis at a hospital in Saudi Arabia: epidemiology, bacterial pathogens and outcome

**DOI:** 10.4103/0256-4947.57164

**Published:** 2009

**Authors:** Jaffar A. Al-Tawfiq, Ismail Sufi

**Affiliations:** a From the Internal Medicine Services Division, Saudi Aramco Medical Services Organization, Dhahran Health Center, Saudi Arabia; b From the Cardiology Services Division, Saudi Aramco Medical Services Organization, Dhahran Health Center, Saudi Arabia

## Abstract

**BACKGROUND AND OBJECTIVE:**

Data on infective endocarditis prevalence, epidemiology and etiology from Saudi Arabia and the Gulf region are sparse. We undertook this study to describe the pattern and the causative agents of endocarditis at a hospital in Saudi Arabia.

**METHODS:**

We conducted a retrospective analysis of all reported endocarditis cases at the Dhahran Health Center from January 1995 to December 2008.

**RESULTS:**

Of the 83 cases of endocarditis, 54 (65%) were definite endocarditis and the remaining 29 (35%) were possible endocarditis based on the Duke criteria. Patients with definite endocarditis included 39 males and 15 females (ratio of 2.6:1) with a mean age (SD) of 59.7 (18.2) years. Of the definite endocarditis cases, native valve endocarditis occurred in 44 (81.5%) cases of and prosthetic valve endocarditis was observed in 10 (18.5%). The most commonly involved valves were mitral (n=24; 44.4%) and aortic (n=20; 39.2%). The most common organisms were *S aureus* (n=23; 42.6%), *Enterococcus faecalis* (n=12; 22.2%) and viridans streptococci (n=9; 16.7%). Surgical intervention was required in 17 (31.4%) cases and the in-hospital mortality rate was 29.4% (n=15). Of all the patients, 3 (5.5%) had embolic stroke as a complication.

**CONCLUSION:**

Native valve endocarditis is the predominant type of endocarditis. The patients were older adults and the most common organisms were *S aureus, E faecalis* and viridans streptococci.

Infective endocarditis (IE) is associated with high morbidity and mortality rates. The current reported in-hospital mortality rate for patients with IE is 15% to 20% with a 1-year mortality rate approaching 40%.^1^ Over the past 20 years, significant changes in the demographic characteristics of IE were reported, including a shift in the age group and a change in the causative organisms.^1^,^2^ A single report suggested that *S aureus* was the more common etiological agent of IE in children.^3^

Data on IE prevalence, epidemiology and etiology from Saudi Arabia and the Gulf region are lacking. Apart from case reports of endocarditis, there is only one study addressing the epidemiology of endocarditis in Saudi Arabia.^4^ Hence, we undertook this study to describe the clinical, microbiological and echocardiographic characteristics as well as the complications of endocarditis in a Saudi Arabian hospital.

## METHODS

This was a retrospective analysis of cases of endocarditis from January 1995 to December 2008 at Saudi Aramco Medical Services Organization (SAMSO). SAMSO provides medical care for Saudi Aramco employees and their dependents: spouses, children and parents (currently, approximately 370 000 individuals are eligible for medical care). The main hospital, Dhahran Health Center (DHC) has a capacity of 380 beds. Admissions to the hospital cover a whole range of patients and include general ward admissions, intensive care and patients receiving chemotherapy for hematological and solid organ malignancy. There are five critical care units including an open heart surgery unit. Transesophageal echocardiography is available. At DHC, medical therapy of patients with IE followed the guidelines of the American Heart Association.^5^

IE cases were retrieved from the computerized database of the health information unit. The case definitions of endocarditis were based on the modified Duke criteria and cases were classified as definite or possible cases accordingly.^6^ We collected the following data: age, gender, predisposing factors, the causative organism, the presence or absence of vegetations on the echocardiogram, the involved cardiac valves, surgical intervention, the indication for surgery, and the 30-day mortality. The data was processed using *S* tatistical Package for the Social Sciences SPSS (10.0). The study was approved by the public relations department in accordance with Saudi Aramco Medical Services Organization policy and procedure (approval number 9-1796).

## RESULTS

A total of 83 cases of endocarditis were identified and 54 (66%) cases were classified as definite endocarditis. The remaining 29 (35%) were possible endocarditis (based on the Duke criteria) and thus were excluded from further analysis. For patients with definite endocarditis, there were 39 males and 15 females (ratio of 2.6:1) with a mean age (SD) of 59.7(18.2) years. The overall incidence of endocarditis was 15.6 per 100 000 discharges during the study period.

Native valve endocarditis occurred in 44 (81.5%) and prosthetic valve endocarditis was observed in 10 (18.5%). Four patients (7.4%) were thought to have healthcare-associated endocarditis. The most commonly involved valves were the mitral (n=24; 44.4%) and aortic valves (n=20; 39.2%) (Table 1). The most common organisms were *S aureus* (n=23; 42.6%), *Enterococcus faecalis* (n=12; 22.2%) and viridans streptococci (n=9; 16.7%) (Table 2). Transthoracic echocardiography (TTE) showed vegetation in 37 (72.5%) and TEE showed vegetation in all the patients who had TEE (n=32) (Table 3). Moreover, TEE detected vegetation in 14 (27.7%) patients whom TTE was negative or questionable (Table 3).

**Table 1:** Involved cardiac valves based on the echo cadiographic findings.

Involved value	Number	%
Prosthetic valve endocarditis	10	18.5
Aortic valve bioprosthesis	3	5.6
Aortic valve bioprosthesis, mitral valve	1	1.9
Aortic mechanical	1	1.9
Tricuspid	1	1.9
Mitral valve bioprosthesis	4	7.4
Native valve endocarditis	44	81.5
Aortic valve, mitral valve	3	5.6
Tricuspid valve	4	7.4
Aortic	17	31.5
Mitral valve	20	37.0

**Total**	**54**	**100.0**

**Table 2:** Causative organisms of endocarditis.

Blood culture	Number	%
Gram negative bacteria	5	9.3
*Brucella*	1	1.9
*Enterobacter aerogenes*	1	1.9
ESBL-producing *klebsiella*	1	1.9
*Pseudomonas aeruginosa*	2	3.7
Gram positive bacteria	49	90.7
*Staphylococcus aureus*	21	38.9
MRSA	2	3.7
Coagulase-negative *staphylococcus*	4	7.4
Viridans streptococci	9	16.7
*Enterococcus faecalis*	12	22.2
Beta-hemolytic *streptococcus*	1	1.9

**Total**	**54**	**100.0**

ESBL: extended-spectrum beta-lactamase, MRSA: methicillin-resistant *S aureus*

**Table 3:** Findings of transthoracic echocardiography (TTE) and transoesophageal echocardiography (TEE) in patients with definite infective endocarditis.

			TEE	
		Positive	Not done	Total
TTE	Positive	18	19	37
	Negative	11	0	11
	Could not be excluded	3	0	3

**Total**		**32**	**19**	**51**

Surgical intervention was required in 17 (33.3%). Of all cases who had surgical intervention, 9 (53%) were due to acute valvular rupture or perforation, 5 (29.4%) due to large vegetations (>10 mm) and two (11.6%) had perivalvular abscess. The in-hospital mortality rate was 29.4% (n=15). The highest mortality rate among patients with *S aureus* endocarditis was 43.7% (7/16 cases) compared to 26.3% (9/35 cases) in other cases (*P* =.221). Of all the patients, 3 (5.5%) had embolic stroke as a complication.

## DISCUSSION

The epidemiology of IE has shown some changes in the last 30 years.^7^ These changes have included the involvement of older age groups and an increased incidence of *S aureus* and gram-negative bacilli. The overall incidence of endocarditis was 15.6 per 100 000 discharges during the study period. Similarly, the incidence rate was 14.6 cases per 100 000 hospital admissions from National Guard Hospital in Riyadh.^4^ Thus based on these two studies, the rate of definite endocarditis in Saudi Arabia is estimated to be about 15 cases per 100 000 admissions/discharges.

The mean age of patients with endocarditis was between 28 and 32 years in studies from Saudi Arabia, Kuwait, India and Tunisia.^4^,^8^–^10^ In contrast, the mean age of the patients in the current study was 58.5 years. This finding is in agreement with recent reports from the United States and Europe. In those studies, 50% or more of endocarditis cases occurred in patients over the age of 60 years, and the median age was 67 years.^11^–^13^ The possible explanations for such a shift are the increasing number of elderly people in the general population and the reduction in the incidence of rheumatic heart disease. The former is particularly true in our hospital with increasing number of geriatric patients.

The most commonly involved valves were the mitral and aortic. This finding is similar to an earlier study from Saudi Arabia where the mitral valve alone was involved in 45.6% and aortic valve in 21.3%.^4^ Similarly, isolated aortic and mitral valve infections occurred in 39% and 21%, respectively.^14^ Transesophageal echocardiography (TEE) detected vegetation in 27.7% patients whom TEE was negative or questionable. TEE demonstrates greater sensitivity compared to TTE in the detection of vegetation in two studies (100% versus 63% and 94% versus 44%, respectively).^15^,^16^

We observed that 42.6% of IE cases were due to *S aureus.* Similarly in a study from Saudi Arabia, *S aureus* accounted for 33% of all the cases.^4^ The International Collaboration on Endocarditis-Merged Database (ICE-MD) also showed that of all patients with native valve endocarditis, 34% had *S aureus* infection.^17^ A similar finding was reported by other investigators and *S aureus* accounted for 50% of endocarditis.^18^ However, this finding is in sharp contrast to a study from Kuwait in 1985-1988 where *Streptococcus viridans* was the commonest infective organism.^10^ Another study from Lebanon showed that 51% of endocarditis cases were due to *Streptococcus* spp.^19^ This difference is related to the time of the study since the current study was done at a later time period.

In a study from National Guard Hospital in Riyadh, methicillin-resistant *S aureus* (MRSA) accounted for 4% of the endocarditis cases.^4^ Similarly, we observed that MRSA endocarditis occurred in 3.7% of all cases. This finding is much lower than those reported in a study from the USA where MRSA endocarditis was seen in 14.8% of patients^17^ and from Lebanon where MRSA endocarditis occurred in 7% of all cases.^19^ The difference may be related to the lower rate of MRSA in our hospital where it constituted 6% of all *S aureus* isolates.^20^ Viridans streptococci constituted 16.7% of the cases and we reported previously a case of endocarditis due to *Granulicatella elegans*.^21^

Our results showed that 33.3% of the cases of IE have required surgical intervention. The indications for surgery were acute valvular rupture or perforation, the presence of large vegetations, and perivalvular abscess, according to the published guidelines.^22^ The surgical rate was lower than the 44.7% rate reported from another study from Saudi^4^ and a 50% rate from Tunisia.^9^ In the International Collaboration on Endocarditis-Prospective Cohort Study, surgical therapy was required in 48.2%.^13^ The high rate of surgical interventions indicates that the threshold for early surgical treatment is lower than before.^13^ It is shown that early surgery may be critical in improving survival in patients with definite IE.^23^–^25^

*S aureus* endocarditis carries a high mortality rate. In the current study, the mortality rate of *S aureus* endocarditis was 29.4%, which was not statistically different than other IE cases. In contrast, in a large study of *S aureus* endocarditis, patients with *S aureus* were more likely to die (20% vs. 12%) than other patients.^18^ In the International Collaboration on Endocarditis–Prospective Cohort Study, in-hospital mortality was 17.7%.^13^ In another study, *S aureus* IE was associated with a higher 1-year mortality rate (43.9% vs. 32.5%; *P*=.04).^1^ Other studies showed a high mortality rate of *S aureus* endocarditis of at least 40%.^26^–^28^ However, most recent studies still show a high mortality rate, approaching 34%.^29^

There are a few limitations of the current study, as it is retrospective and uncontrolled in design. Second, it involved patients from one institute and may have referral bias. Third, the number of patients is relatively low in comparison to international registries, but is similar to single institution reports. The study shows that developing a national registry for endocarditis throughout Saudi Arabia is an ambition and would be of particular help to health care.

In conclusion, IE remains a serious disease with high morbidity and mortality rates. In addition, IE has shifted to a disease in older adults with a high rate of *S aureus* infections. Geographic differences in the presentation, microbial etiology, treatment, and outcome of patients with IE need to be taken into consideration.^13^
